# Postoperative Complications Following Total Knee Arthroplasty in Patients with Chronic Lymphocytic Leukemia

**DOI:** 10.3390/jcm15156078

**Published:** 2026-08-05

**Authors:** Brandon Wood, Sri Tummala, Dharani Rohit Thota, Senthil Sambandam

**Affiliations:** 1 School of Medicine, University of Texas Southwestern Medical Center, Dallas, TX 75390, USA; brandon.wood@utsouthwestern.edu; 2 Department of Medical Education, Texas A&M School of Medicine, Baylor University Medical Center, 3500 Gaston Avenue, 6-Roberts, Dallas, TX 75246, USA; sr1@tamu.edu; 3 Department of Orthopedic Surgery, University of Texas Southwestern Medical Center, Dallas, TX 75390, USA; dharanirohit.thota@utsouthwestern.edu

**Keywords:** TKA, CLL, postoperative complications

## Abstract

**Background/Objectives:** Chronic lymphocytic leukemia (CLL), the most common leukemia in adults, predominantly affects the aging population. With improved survival from modern targeted therapies, a growing number of CLL patients are living long enough to become candidates for quality-of-life-improving procedures such as elective total knee arthroplasty (TKA). However, the impact of CLL on postoperative outcomes remains poorly understood. **Methods:** A retrospective cohort analysis was performed using the TriNetX Research Network. Adult patients who underwent primary TKA were identified and divided into two cohorts. CLL and non-CLL control, and 1:1 propensity score matching was performed on demographics and comorbidities. Medical complications were assessed at 30 and 90 days, and implant-related complications at 1 and 3 years postoperatively. Relative risks (RRs) with 95% confidence intervals (CIs) were calculated. Within the CLL cohort, a Cox proportional hazards model was used to identify preoperative laboratory values, medications, and comorbid diagnoses associated with complication development. **Results:** After matching, 2054 patients, 1027 per cohort, were analyzed. Patients with CLL demonstrated significantly higher risks of postoperative pain at both 30 days (*p* < 0.001) and 90 days (*p* < 0.001), as well as transfusion (*p* = 0.035) and acute kidney injury (*p* = 0.036) at 90 days. At 1 year, the CLL cohort had elevated risks of periprosthetic joint infection (PJI; *p* = 0.015) and arthrofibrosis (*p* = 0.036), and these differences persisted at 3 years (PJI *p* = 0.033; arthrofibrosis *p* = 0.006). In the hazards model, preoperative hemoglobin < 8 g/dL (HR: 1.75, 95% CI: 1.27–2.41, *p* < 0.001), long-term anticoagulant or antiplatelet use (HR: 1.43, 95% CI: 1.13–1.80, *p* = 0.003), chronic kidney disease (CKD; HR: 1.28, 95% CI: 1.04–1.58, *p* = 0.021), and overweight/obesity (HR: 1.23, 95% CI: 1.00–1.50, *p* = 0.047) were independently associated with short-term complications. Chronic opioid use was found to be associated with an increased risk of long-term complications (HR: 2.80, 95% CI: 1.49–5.24, *p* = 0.001). **Conclusions:** Patients with CLL undergoing TKA had higher observed risks of both early medical and late implant-related complications. These findings may reflect the combined effects of CLL-related immune dysfunction, cytopenias, and chronic systemic inflammation, which may impair wound healing, reduce physiologic reserve, and blunt infection clearance. Identification of associated, modifiable preoperative risk factors—anemia, anticoagulant exposure, CKD, obesity, and chronic opioid use—may provide actionable targets for optimization and risk stratification.

## 1. Introduction

Total knee arthroplasty (TKA) is among the most commonly performed elective surgical procedures in the United States and is a mainstay of treatment for end-stage knee osteoarthritis, offering durable pain relief and functional improvement [1]. Recent projections using Centers for Medicare & Medicaid Services data estimate that annual primary TKA volume among Medicare beneficiaries will increase from approximately 1.0 million procedures in 2022 to nearly 2.8 million by 2040, representing an annual growth rate of 5.9% [2]. This projected increase is largely attributable to growth in the elderly population. The number of people in the US over the age of 65 is expected to rise from 58 million in 2022 to 76 million by 2035 and 82 million by 2050 [3]. In the US population over 65, this would represent an increase from 17% in 2022 to 23% in 2050 [4]. Given the aging population of the US, the number of osteoarthritis cases is anticipated to increase by 60–100% by 2050 [5].

Another disease that disproportionately affects the elderly population is chronic lymphocytic leukemia (CLL), the most common form of leukemia in the US [6,7]. While 70–80% of CLL patients are asymptomatic, those who report symptoms often complain of lymphadenopathy, fatigue, frequent infections, fever, and weight loss or experience complications such as anemia, autoimmune disorders, immunodeficiency, and secondary cancers [8,9,10,11,12]. Despite the prevalence of CLL, the introduction of targeted therapies such as Bruton tyrosine kinase (BTK) inhibitors and B-cell lymphoma 2 (BCL2) inhibitors (e.g., venetoclax) has substantially reduced mortality and improved overall survival in this population [7,8]. Osteoarthritis is also highly prevalent among patients with CLL. In a large prospective cohort of newly diagnosed patients, osteoarthritis was found to be the most common comorbid condition, affecting approximately 42% of individuals at the time of diagnosis [13]. With improved life expectancy and a high burden of concomitant osteoarthritis, an increasing number of patients with CLL are likely to become candidates for quality-of-life–improving procedures such as TKA. As a result, further research into the impact of CLL on arthroplasty outcomes is warranted.

Despite the high prevalence of osteoarthritis among patients with CLL, limited research has evaluated the effect of CLL on postoperative outcomes following TKA. Deliwala et al. found that patients with a general diagnosis of cancer were associated with worse outcomes following TKA at the 5-year postoperative mark [14]. A study conducted in 2018 found that patients with hematologic malignancies were at an increased risk of perioperative complications [15]. A more recent study completed using the PearlDiver database examined 90-day medical complications and 2-year implant-related complications and found that TKA patients had a greater risk of developing various medical complications at 90 days and implant-related complications at 2 years [16]. While this study proved informative on the topic, statistical analysis to determine what factors may increase risk of postoperative complications was not included. The present study provides further analysis of the topic by examining medical and implant-related complications using a different database and will expand our understanding of postoperative complications following TKA in the CLL population through the evaluation of pertinent risk factors that influence their development.

## 2. Materials and Methods

A retrospective analysis was conducted using the TriNetX Database (https://trinetx.com, Cambridge, MA, USA, accessed on 7 March 2026), a federated health research platform, to evaluate patients from 2009 through 2025. This database contains deidentified ICD-10 and CPT codes. As the study utilized deidentified data from the TriNetX Research Network, institutional review board approval was not required. Patients who underwent primary TKA were identified, and two cohorts of patients were isolated: patients diagnosed with CLL before TKA (CLL cohort) and patients without a CLL diagnosis (control).

To reduce baseline differences between cohorts, patients with CLL were matched 1:1 to controls using greedy nearest-neighbor propensity score matching without replacement and a caliper width of 0.10. Matching was performed using baseline demographic and clinical characteristics, including age, sex, race, ethnicity, overweight/obesity, smoking status, diabetes mellitus, hypertension, chronic obstructive pulmonary disease, heart failure, use of anticoagulants, and reduced mobility. Covariate balance before and after matching was assessed using standardized mean differences (SMDs), with an SMD < 0.10 considered indicative of adequate balance. Additionally, preoperative leukocyte count was extracted as an indicator of disease burden.

Following matching, the groups were compared to identify common perioperative and short-term postoperative complications at both 30 days and 90 days post TKA. Long-term postoperative complications were identified at 1 year and 3 years postoperative. ICD-10 and CPT codes were used to examine various medical complications and healthcare utilization statistics at 30 days and 90 days postoperative. Implant-related complications and surgical complications were examined at 1 year and 3 years postoperative. All data points are cumulative from the index TKA. Patients were followed from the index procedure until the occurrence of the outcome of interest or the end of the specified follow-up period. The results were compared using relative risk (RR), 95% confidence intervals (CIs), and *p*-values, with a *p*-value less than 0.05 demonstrating statistical significance. All calculations were completed using the software included in the TriNetX program.

After analyzing the matched data, the CLL cohort was further stratified into those who experienced at least one of the short-term or long-term complications examined at 90 days and 3 years postoperative, respectively, and those who did not experience complications. These two groups were then compared using a Cox proportional hazard model to identify whether differences in age, sex, blood counts, medications, diagnoses, or common CLL treatments were associated with the composite short-term and long-term outcomes. Lab data collected up to a year prior to their procedure was included in the hazard model, while the codes for various medications, diagnoses, and common CLL treatments were analyzed for use at any time before the operation. Covariates included in the Cox proportional hazards models were selected based on established clinical relevance and prior literature demonstrating their association with postoperative complications following TKA and outcomes in patients with CLL. The proportional hazard assumption was evaluated using the proportionality test available within the TriNetX analytics platform. The reported measures included hazard ratio (HR), 95% CI, and two-tailed *p*-value (*p* > |z|). Statistical significance was defined as a two-sided *p*-value < 0.05. These calculations were also completed using the statistical software included in the TriNetX program.

The ICD-10 and CPT codes used to define the cohorts and the postoperative complications can be found in the Supplementary Material (Table S1).

## 3. Results

### 3.1. Population Statistics

Prior to matching, the CLL cohort contained 1027 patients while the control cohort contained 445,366 patients. Following propensity score matching, the population of the study included 2054 patients, with 1027 in both the CLL and control cohorts. All covariates included in propensity-score matching demonstrated adequate post-match balance, with SMDs below 0.10. The CLL cohort was found to have an increased leukocyte count when compared to the control cohort (*p* < 0.001) [Table 1].

### 3.2. Risk of Short-Term Complications

The CLL cohort was found to have a significantly higher risk of postoperative pain at both 30 days (RR: 1.75, CI: 1.32–2.33) and 90 days (RR: 1.65, CI: 1.27–2.16) postoperative. At 90 days postoperative, this cohort was also found to have an increased risk of transfusion (RR: 1.50, CI: 1.03–2.29) and acute kidney injury (AKI) (RR: 1.73, CI: 1.03–2.90) [Table 2].

### 3.3. Risk of Long-Term Complications

At 1 year postoperative, the CLL cohort demonstrated an increased risk of PJI (RR: 1.80, CI: 1.11–2.91) and arthrofibrosis (RR: 2.00, CI: 1.03–3.87). This trend continued at 3 years postoperative, as risk of both PJI (RR: 1.59, CI: 1.04–2.46) and arthrofibrosis (RR: 2.39, CI: 1.26–4.53) was again found to be significantly elevated [Table 3].

### 3.4. Cox Proportional Hazard Model Analysis

The Cox proportional hazard model for short-term complications at 90 days postoperative identified several independent predictors of complication development within the CLL cohort. Patients with a preoperative hemoglobin less than 8 g/dL within one year of surgery had a significantly higher hazard of developing a short-term complication (HR: 1.75, CI: 1.27–2.41). Long-term or current use of anticoagulant or antiplatelet therapy was similarly associated with elevated risk (HR: 1.43, CI: 1.13–1.80). A diagnosis of chronic kidney disease (CKD) (HR: 1.28, CI: 1.04–1.58) or a documented diagnosis of overweight/obesity (HR: 1.23, CI: 1.00–1.50) at any time prior to surgery also independently increased short-term complication risk [Table 4].

Risk of developing a long-term complication at 3 years postoperative was found to be significant in CLL patients with long-term and current use of opiate analgesic (HR: 2.80, CI: 1.49–5.24) [Table 5].

## 4. Discussion

As the population of patients undergoing TKA continues to expand, understanding how comorbid conditions influence postoperative outcomes remains increasingly important. In the present study, patients with CLL had significantly higher observed rates of both early medical complications and longer-term implant-related complications following TKA compared with matched controls. An association with an increased risk of postoperative pain, transfusion, and AKI was observed in the early postoperative period, while rates of PJI and arthrofibrosis were elevated at longer-term follow-up. Further analysis of the CLL cohort identified several characteristics associated with increased risk of postoperative complications, including anemia, anticoagulant use, CKD, obesity, and opioid exposure.

Our findings are generally consistent with those reported by Rodriguez et al., who also reported higher rates of both medical and implant-related complications among patients with CLL undergoing TKA compared with matched controls [16]. Specifically, both studies identified increased risks of postoperative transfusion and periprosthetic joint infection, supporting the reproducibility of these associations across two large national databases. However, whereas Rodriguez et al. reported elevated risks for several additional postoperative complications, our analysis found significant differences in a more limited subset of outcomes. These differences may reflect variations in database composition, matching methodology, coding practices, and outcome ascertainment between PearlDiver and TriNetX rather than true differences in the underlying patient population. Furthermore, the present study extends those findings in several important ways. By leveraging the TriNetX Research Network, we evaluated postoperative outcomes at both 30 and 90 days, as well as 1 and 3 years, providing a more granular assessment of complication timing. Furthermore, we incorporated a Cox proportional hazards analysis within the CLL cohort to identify patient-specific characteristics associated with postoperative complications. This analysis identified potentially modifiable or clinically actionable risk factors that may improve preoperative risk stratification and optimization in this high-risk population.

Chronic lymphocytic leukemia was found to be associated with significantly higher rates of several early postoperative complications following TKA, including postoperative pain, transfusion, and AKI. These findings may be related to the underlying hematologic and immunologic abnormalities associated with CLL. The disease is characterized by impaired immune function, cytopenias, and chronic systemic inflammation, all of which may negatively influence physiologic reserve and postoperative recovery [9,17,18]. Anemia and bone marrow dysfunction, frequently seen in patients with CLL, may contribute to an increased need for perioperative transfusion, while the higher prevalence of medical comorbidities in this population may predispose patients to complications such as AKI [18,19]. Additionally, the increased rate of postoperative pain observed in this cohort may reflect a combination of baseline inflammatory burden and comorbid disease [18,20]. One possible explanation for this finding also included a baseline tolerance of opioids in the CLL population, though this was not supported by the Cox proportional hazards model. Together, these factors highlight how the systemic effects of CLL may contribute to increased vulnerability to early postoperative complications following arthroplasty.

In addition to early postoperative complications, CLL was also correlated with a significantly increased risk of long-term implant-related complications following TKA. The elevated risk of PJI may be attributable to the immunologic dysfunction associated with CLL. Patients with this disease commonly exhibit impaired humoral immunity and hypogammaglobulinemia, which can reduce the body’s ability to mount an effective immune response to bacterial pathogens and increase susceptibility to postoperative infections [21,22]. The increased incidence of arthrofibrosis observed may also be related to the systemic inflammatory state associated with CLL as well as the increased postoperative pain identified in this study, as both may negatively impact early mobilization and rehabilitation following surgery [18]. Although the absolute increase in PJI risk was modest, the clinical consequences of infection after TKA are substantial, often requiring prolonged antibiotic therapy, reoperation, or revision arthroplasty. Recognition of this elevated risk may therefore inform perioperative counseling and postoperative monitoring.

In addition to identifying increased complication rates, this study sought to evaluate factors within the CLL cohort that may predispose patients to adverse outcomes following TKA. The Cox proportional hazards model identified several patient characteristics associated with an increased risk of postoperative complications. Anemia within a year of the procedure was associated with a significantly higher hazard of developing postoperative complications. This association may reflect decreased physiologic reserve and impaired tissue oxygenation that can negatively affect postoperative recovery and wound healing [23]. Long-term and current use of anticoagulant and antithrombotic medications was also correlated with increased complication risk, potentially reflecting an elevated perioperative bleeding risk [24]. These findings are consistent with the elevated rate of transfusion in the postoperative period demonstrated in this study. Additionally, CKD and overweight or obesity were identified as potential risk factors for postoperative complications, conditions that have previously been associated with systemic inflammation and increased susceptibility to complications following joint arthroplasty [25,26,27,28]. Taken together, these findings suggest that both disease-related factors and underlying patient comorbidities may contribute to postoperative risk in this population and highlight the importance of careful preoperative evaluation and risk stratification when managing patients with CLL undergoing TKA. Identification of these modifiable and nonmodifiable risk factors may help surgeons individualize perioperative risk assessment and facilitate multidisciplinary optimization before surgery, particularly among patients with multiple medical comorbidities.

Long-term opioid use prior to surgery was also found to be associated with an increased risk of postoperative complications within the CLL cohort following TKA. This finding is clinically important, as preoperative opioid exposure has been increasingly recognized as a marker of higher baseline pain burden, reduced functional status, and greater overall medical complexity in patients undergoing arthroplasty [29,30]. Chronic opioid use may also contribute to opioid tolerance and opioid-induced hyperalgesia, both of which can complicate postoperative pain control and negatively affect early mobilization and participation in rehabilitation [31].

These findings have important clinical implications for the perioperative management of patients with CLL undergoing TKA. Given the increased risks observed in this population, careful preoperative evaluation and optimization of potentially modifiable factors may be beneficial. Correction of preoperative anemia, optimization of renal function, and management of obesity may help reduce perioperative risk, while perioperative management of anticoagulant and antithrombotic therapy is warranted to balance bleeding and thrombotic risk. Additionally, recognition of chronic opioid use as a marker of increased surgical risk may allow for targeted interventions, including multimodal pain optimization and enhanced postoperative rehabilitation planning. Collectively, these strategies may help optimize outcomes and support more informed shared decision-making when counseling patients with CLL regarding expectations and risks associated with TKA.

### Limitations

This study should be interpreted in the context of several limitations. First, the use of a large electronic health record database is subject to potential coding inaccuracies and misclassification bias inherent to ICD-10 and CPT-based data capture. Specifically, diagnoses such as postoperative pain and reduced mobility rely on provider documentation and coding practices, which may vary across clinicians and institutions. Consequently, these outcomes are susceptible to measurement variability and potential misclassification and should therefore be interpreted with appropriate caution.

Furthermore, important clinical details such as disease stage, duration of disease, cytogenetic risk, treatment status, and other markers of CLL severity were unavailable. Because CLL is a clinically heterogeneous disease, these factors may substantially influence immune function, hematologic status, and overall physiologic reserve, all of which could affect perioperative risk. Consequently, our findings should be interpreted as reflecting outcomes across a broad population of patients with CLL rather than within specific disease subgroups.

Additionally, laboratory data were not available for all patients and were limited to values recorded within the TriNetX network. This may not have been random, as patients with more active disease, greater comorbidity burden, or more frequent healthcare utilization are more likely to undergo laboratory testing than healthier individuals. Consequently, the analyzed cohort in the Cox proportional hazards analyses may overrepresent patients with greater healthcare utilization or more active disease. Furthermore, patient-reported outcome measures such as preoperative pain severity were not available within the database. Consequently, baseline differences in pain between cohorts could not be assessed and may have influenced the observed rates of postoperative pain. Also, as complete follow-up was not required for inclusion in the cumulative risk analyses, patients with more recent index procedures may have had less opportunity to experience long-term outcomes, potentially resulting in underestimation of 1- and 3-year complication rates.

Similarly, granular operative data including surgical technique, surgeon experience, hospital volume, implant characteristics, intraoperative details, and postoperative rehabilitation protocols were not available, which may also influence postoperative outcomes. Although propensity score matching and multivariable modeling were used to reduce confounding, the possibility of residual confounding from unmeasured variables remains.

The Cox proportional hazards models evaluated composite short-term and long-term postoperative outcomes; therefore, the observed associations were driven primarily by the more frequently occurring complications within each composite endpoint. Given the relatively small sample size, separate Cox analyses for individual postoperative complications were not feasible. Consequently, the identified risk factors should be interpreted as predictors of overall postoperative complications rather than of specific complications with relatively low event rates.

Finally, mortality was not evaluated in the present study, and death may have acted as a competing event for long-term postoperative complications such as prosthetic joint infection, arthrofibrosis, or revision surgery. Consequently, patients who died before experiencing these outcomes may not have been captured, potentially influencing estimates of long-term complication rates. Future studies incorporating competing-risk analyses are warranted to better characterize these relationships.

Despite these limitations, the large sample size, multicenter representation, and inclusion of laboratory and risk factor data provide clinically meaningful insights into postoperative risk stratification in this population.

## 5. Conclusions

Patients with CLL undergoing TKA had higher observed risks of both short-term medical complications, including postoperative pain, transfusion, and acute kidney injury, and long-term implant-related complications (PJI and arthrofibrosis) compared with matched controls. In addition, this study identified several potentially or clinically actionable patient-specific factors, including preoperative anemia, anticoagulant use, chronic kidney disease, obesity, and chronic opioid exposure, that are associated with an elevated risk of postoperative complications. These findings highlight the importance of careful preoperative risk assessment and optimization in this population and may inform risk stratification, shared decision-making, and tailored perioperative planning. Further prospective studies are warranted to better define optimal management strategies and improve outcomes in patients with CLL undergoing TKA.

## Figures and Tables

**Table 1:** Post-match demographic data.

	Post-Match Demographics						
	CLL		Control				
Characteristic	n	Mean or %	n	Mean or %	SMD Prior to Matching	SMD After Matching	*p*-Value
Age at Index	1027	71.9 ± 8.3	1027	71.9 ± 8.4	0.604	0.001	0.981
Sex							
Male	522	51%	520	51%	0.234	0.004	0.930
Female	505	49%	507	49%	0.232	0.002	0.965
Race and Ethnicity							
Hispanic or Latino	16	2%	15	2%	0.159	0.008	0.856
White	930	91%	932	91%	0.349	0.007	0.880
Black or African American	51	5%	54	5%	0.254	0.013	0.764
Other or Unknown	30	3%	26	3%	0.034	0.035	0.588
Diagnosis							
Tobacco Use	28	3%	25	2%	0.014	0.018	0.676
Diabetes Mellitus	281	27%	279	27%	0.175	0.004	0.921
Essential (Primary) Hypertension	738	72%	737	72%	0.359	0.002	0.961
Overweight/Obese	353	34%	354	34%	0.106	0.002	0.963
Heart Failure	122	12%	123	12%	0.227	0.003	0.946
COPD	103	10%	101	10%	0.142	0.007	0.883
Reduced Mobility	19	2%	19	2%	0.051	<0.001	1.000
Medication							
Anticoagulants	506	49%	506	49%	0.456	<0.001	1.000
Labs	n with lab		n with lab				
**Leukocyte Count**	**944**	**20 ± 92.7**	**882**	**7.1 ± 2**	**0.134**	**0.196**	**<0.001**

Significant *p*-values are bolded (*p* < 0.05); n with lab refers to number of patients with lab data as reported by TriNetX; COPD = chronic obstructive pulmonary disease.

**Table 2:** Complications at 30 days and 90 days postoperative.

**30 Days**							
	**CLL**		**Control**				
**Complication**	**n**	**%**	**n**	**%**	**RR**	**95% CI**	***p*-Value**
Transfusion	39	4%	25	2%	1.56	(0.95, 2.56)	0.076
Acute Blood Loss Anemia	62	6%	55	5%	1.13	(0.79, 1.60)	0.505
Lower Extremity DVT	20	2%	23	2%	0.87	(0.48, 1.57)	0.644
Acute Kidney Injury	31	3%	18	2%	1.72	(0.97, 3.06)	0.060
Pneumonia	-	-	-	-	-	-	-
Atelectasis	-	-	-	-	-	-	-
Postoperative Infection	-	-	-	-	-	-	-
**Postoperative Pain**	**121**	**12%**	**69**	**7%**	**1.75**	**(1.32, 2.33)**	**<0.001**
Readmission	142	14%	128	12%	1.11	(0.89, 1.39)	0.361
Emergency Department Visits	86	8%	69	7%	1.25	(0.92, 1.69)	0.156
**90 Days**							
	**CLL**		**Control**				
**Complication**	**n**	**%**	**n**	**%**	**RR**	**95% CI**	***p*** **-Value**
**Transfusion**	**63**	**6%**	**42**	**4%**	**1.50**	**(1.03, 2.29)**	**0.035**
Acute Blood Loss Anemia	69	7%	62	6%	1.11	(0.80, 1.55)	0.528
Lower Extremity DVT	29	3%	29	3%	1.00	(0.60, 1.66)	1.000
**Acute Kidney Injury**	**38**	**4%**	**22**	**2%**	**1.73**	**(1.03, 2.90)**	**0.036**
Pneumonia	22	2%	15	1%	1.47	(0.77, 2.81)	0.246
Atelectasis	13	1%	12	1%	1.08	(0.50, 2.36)	0.841
Postoperative Infection	14	1%	11	1%	1.27	(0.58, 2.79)	0.546
**Postoperative Pain**	**129**	**12%**	**78**	**8%**	**1.65**	**(1.27, 2.16)**	**<0.001**
Readmission	189	18%	171	17%	1.11	(0.92, 1.33)	0.297
Emergency Department Visits	127	12%	117	11%	1.09	(0.86, 1.37)	0.500

Significant *p*-values are bolded (*p* < 0.05); values greater than 0 and less than or equal to 10 are not reported by TriNetX as indicated using “-“ in the table; DVT = Deep vein thrombosis.

**Table 3:** Complications at 1 year and 3 years postoperative.

**1 Year**							
	**CLL**		**Control**				
**Complication**	**n**	**%**	**n**	**%**	**RR**	**95% CI**	***p*** **-Value**
Mechanical Complications	37	4%	36	3%	1.03	(0.66, 1.61)	0.905
Revision TKA	14	1%	11	1%	1.27	(0.58, 2.79)	0.546
Periprosthetic Fracture	-	-	-	-	-	-	-
Prosthetic Dislocation	-	-	-	-	-	-	-
**Periprosthetic Joint Infection**	**45**	**4%**	**25**	**2%**	**1.80**	**(1.11, 2.91)**	**0.015**
Manipulation Under Anesthesia	26	3%	17	2%	1.53	(0.84, 2.80)	0.166
**Arthrofibrosis**	**26**	**3%**	**13**	**1%**	**2.00**	**(1.03, 3.87)**	**0.036**
**3 Years**							
	**CLL**		**Control**				
**Complication**	**n**	**%**	**n**	**%**	**RR**	**95% CI**	***p*** **-Value**
Mechanical Complications	47	5%	49	5%	0.96	(0.64, 1.44)	0.834
Revision TKA	19	2%	18	2%	1.06	(0.56, 2.00)	0.868
Periprosthetic Fracture	-	-	-	-	-	-	-
Prosthetic Dislocation	12	1%	12	1%	1.00	(0.45, 2.22)	1.000
**Periprosthetic Joint Infection**	**51**	**5%**	**32**	**3%**	**1.59**	**(1.04, 2.46)**	**0.033**
Manipulation Under Anesthesia	29	3%	17	2%	1.71	(0.94, 3.09)	0.074
**Arthrofibrosis**	**31**	**3%**	**13**	**2%**	**2.39**	**(1.26, 4.53)**	**0.006**

Significant *p*-values are bolded (*p* < 0.05); values greater than 0 and less than or equal to 10 are not reported by TriNetX, as indicated using “-“ in the table; TKA = Total knee arthroplasty.

**Table 4:** Cox proportional hazards model at 90 days postoperative.

Covariate		Hazard Ratio	95% CI	*p* > |z|
Male Sex		1.05	(0.86, 1.27)	0.659
Age at Index		1.01	(0.99, 1.02)	0.072
**Hemoglobin**	**<8 g/dL**	**1.75**	**(1.27, 2.41)**	**<0.001**
	8–12 g/dL	0.48	(0.11, 2.08)	0.323
	12–18 g/dL	0.86	(0.58, 1.28)	0.458
Platelets	<150,000/µL	0.86	(0.44, 1.70)	0.671
	150,000–400,000/µL	1.06	(0.80, 1.40)	0.707
	400,000–600,000/µL	1.43	(0.97, 2.10)	0.073
Leukocytes	<4500/µL	0.81	(0.24, 2.69)	0.723
	4500–11,000/µL	1.08	(0.86, 1.36)	0.508
	11,000–20,000/µL	0.82	(0.67, 1.00)	0.054
	>20,000/µL	1.21	(0.96, 1.54)	0.112
Diabetes Mellitus		1.01	(0.82, 1.24)	0.922
Tobacco Use		0.89	(0.54, 1.45)	0.631
COPD		1.12	(0.86, 1.44)	0.405
**Chronic Kidney Disease**		**1.28**	**(1.04, 1.58)**	**0.021**
**Overweight and Obesity**		**1.23**	**(1.00, 1.50)**	**0.047**
Abnormalities of Gait and Mobility		1.08	(0.87, 1.35)	0.470
Chemotherapy		0.83	(0.65, 1.05)	0.113
Stem Cell Transplant		0.78	(0.39, 1.58)	0.496
BTK Inhibitors		0.86	(0.54, 1.36)	0.521
Venetoclax		1.63	(0.83, 3.19)	0.156
Long Term Use of Opiate Analgesic		0.76	(0.58, 1.01)	0.055
**Long Term Use of Anticoagulants and Antithrombotics/Antiplatelets**		**1.43**	**(1.13, 1.80)**	**0.003**

Significant *p*-values are bolded (*p* < 0.05); COPD = chronic obstructive pulmonary disease and BTK = Bruton tyrosine kinase.

**Table 5:** Cox proportional hazards model at 3 years postoperative.

Covariate		Hazard Ratio	95% CI	*p* > |z|
Male Sex		1.16	(0.78, 1.71)	0.474
Age at Index		0.99	(0.97, 1.02)	0.779
Hemoglobin	<8 g/dL	0.98	(0.55, 1.73)	0.939
	8–12 g/dL	-	-	-
	12–18 g/dL	-	-	-
Platelets	<150,000/µL	0.97	(0.28, 3.41)	0.966
	150,000–400,000/µL	1.69	(0.94, 3.04)	0.082
	400,000–600,000/µL	1.21	(0.65, 2.26)	0.547
Leukocytes	<4500/µL	0.88	(0.10, 7.57)	0.910
	4500–11,000/µL	1.09	(0.71, 1.67)	0.698
	11,000–20,000/µL	1.01	(0.68, 1.49)	0.977
	>20,000/µL	0.89	(0.56, 1.40)	0.611
Diabetes Mellitus		0.97	(0.65, 1.46)	0.882
Tobacco Use		0.39	(0.14, 1.46)	0.074
COPD		1.02	(0.62, 1.69)	0.926
Chronic Kidney Disease		0.89	(0.57, 1.40)	0.607
Overweight and Obesity		0.86	(0.57, 1.29)	0.464
Abnormalities of Gait and Mobility		0.94	(0.60, 1.48)	0.795
Chemotherapy		1.24	(0.80, 1.94)	0.339
Stem Cell Transplant		0.37	(0.09, 1.55)	0.174
BTK Inhibitors		1.05	(0.52, 2.14)	0.886
Venetoclax		1.99	(0.64, 6.14)	0.232
**Long Term Use of Opiate Analgesic**		**2.80**	**(1.49, 5.24)**	**0.001**
Long Term Use of Anticoagulants and Antithrombotics/Antiplatelets		1.25	(0.74, 2.11)	0.414

Significant *p*-values are bolded (*p* < 0.05); hemoglobin values of 8–12 g/dL and 12–18 g/dL were unable to be assessed due to a small proportion of the population with those lab values; COPD = chronic obstructive pulmonary disease and BTK = Bruton tyrosine kinase.

## Data Availability

Restrictions apply to the availability of these data. Data were obtained from TriNetX and are available from the authors with permission or access to TriNetX.

## Supplementary Materials

The following supporting information can be downloaded at: https://www.mdpi.com/article/10.3390/jcm15156078/s1, Table S1: Codes Used to Define Cohorts, Covariates, Procedures, and Postoperative Outcomes in TriNetX.

## Abbreviations

The following abbreviations are used in this manuscript:

BTK	Bruton tyrosine kinase
CI	Confidence interval
CLL	Chronic lymphocytic leukemia
COPD	Chronic obstructive pulmonary disease
DVT	Deep vein thrombosis
ER	Emergency room
HR	Hazard ratio
RR	Relative risk
TKA	Total knee arthroplasty

## References

[1] Li J.W., Ma Y.S., Xiao L.K. (2019). Postoperative Pain Management in Total Knee Arthroplasty. Orthop. Surg..

[2] Jones C.M., Potluri A.S., Federico V.P., Nie J.W., Forlenza E.M., Serino J., Della Valle C.J. (2025). Trends in Medicare Arthroplasty Procedure Volume: Projecting from 2025 to 2040. J. Arthroplast..

[3] United States Census Bureau (2023). 2023 National Population Projections Tables: Main Series.

[4] Jones C.H., Dolsten M. (2024). Healthcare on the Brink: Navigating the Challenges of an Aging Society in the United States. npj Aging.

[5] GBD 2021 Osteoarthritis Collaborators (2023). Global, Regional, and National Burden of Osteoarthritis, 1990–2020 and Projections to 2050: A Systematic Analysis for the Global Burden of Disease Study 2021. Lancet Rheumatol..

[6] Gribben J.G. (2010). Chronic Lymphocytic Leukemia: Planning for an Aging Population. Expert Rev. Anticancer Ther..

[7] Siegel R.L., Kratzer T.B., Giaquinto A.N., Sung H., Jemal A. (2025). Cancer Statistics, 2025. CA Cancer J. Clin..

[8] Shadman M. (2023). Diagnosis and Treatment of Chronic Lymphocytic Leukemia: A Review. JAMA.

[9] Binder A.F., Gabrilove J.L. (2019). Chronic Lymphocytic leukemia. Oncology.

[10] Elhadary M., Elshoeibi A.M., Badr A., Elsayed B., Metwally O., Elshoeibi A.M., Mattar M., Alfarsi K., AlShammari S., Alshurafa A. (2023). Revolutionizing Chronic Lymphocytic Leukemia Diagnosis: A Deep Dive into the Diverse Applications of Machine Learning. Blood Rev..

[11] Gauthier M. (2022). Chronic Lymphocytic Leukemia. Rev. Med. Internet.

[12] Molica S., Mirabelli R., Molica M., Levato L., Mauro F.R., Foà R. (2011). Clinical Relevance and Treatment of Nonautoimmune Anemia in Chronic Lymphocytic Leukemia. Cancer Manag. Res..

[13] Strati P., Parikh S.A., Chaffee K.G., Achenbach S.J., Call T.G., Ding W., Leis J.F., Kenderian S.S., Schwager S.M., Shanafelt T.D. (2017). Relationship Between Co-Morbidities at Diagnosis, Survival and Ultimate Cause of Death in Patients with Chronic Lymphocytic Leukaemia (CLL): A Prospective Cohort Study. Br. J. Haematol..

[14] Deliwala I.H., Liu A.T., Bank N.C., Reynolds C.A., Narayanan A.S. (2026). The Five-Year Outcomes of Total Knee Arthroplasty in Patients Who Have Cancer. J. Arthroplast..

[15] Newman J.M., Wagner T.C., Naziri Q., Ponzio D.Y., Cooper H.J., Shah R.P., Courtney P.M., Westrich G.H. (2018). Hematologic Malignancies Are Associated with Adverse Perioperative Outcomes Following Total Knee Arthroplasty. J. Knee Surg..

[16] Rodriguez H.C., Mekkawy K.L., Rust B.D., Gomez O., Corces A., Roche M.W. (2025). Medical- and Implant-Related Complications Following Total Joint Arthroplasty in Patients Who Have Chronic Lymphocytic Leukemia. J. Arthroplast..

[17] Tsang M., Parikh S.A. (2017). A Concise Review of Autoimmune Cytopenias in Chronic Lymphocytic Leukemia. Curr. Hematol. Malig. Rep..

[18] Caligaris-Cappio F. (2011). Inflammation, the Microenvironment and Chronic Lymphocytic Leukemia. Haematologica.

[19] Zent C.S., Kay N.E. (2010). Autoimmune Complications in Chronic Lymphocytic Leukaemia (CLL). Best Pract. Res. Clin. Haematol..

[20] Villavicencio A., Solans M., Zacarías-Pons L., Vidal A., Puigdemont M., Roncero J.M., Saez M., Marcos-Gragera R. (2021). Comorbidities at Diagnosis, Survival, and Cause of Death in Patients with Chronic Lymphocytic Leukemia: A Population-Based Study. Int. J. Environ. Res. Public Health.

[21] Grywalska E., Zaborek M., Łyczba J., Hrynkiewicz R., Bębnowska D., Becht R., Sosnowska-Pasiarska B., Smok-Kalwat J., Pasiarski M., Góźdź S. (2020). Chronic Lymphocytic Leukemia-Induced Humoral Immunosuppression: A Systematic Review. Cells.

[22] Parikh S.A., Leis J.F., Chaffee K.G., Call T.G., Hanson C.A., Ding W., Chanan-Khan A.A., Bowen D., Conte M., Schwager S. (2015). Hypogammaglobulinemia in Newly Diagnosed Chronic Lymphocytic Leukemia: Natural History, Clinical Correlates, and Outcomes. Cancer.

[23] Zhang F.Q., Yang Y.Z., Li P.F., Ma G.R., Zhang A.R., Zhang H., Guo H.Z. (2024). Impact of Preoperative Anemia on Patients Undergoing Total Joint Replacement of Lower Extremity: A Systematic Review and Meta-Analysis. J. Orthop. Surg. Res..

[24] Wagner J., Lock J.F., Kastner C., Hoffmann M., Gutt C.N. (2019). Perioperative Management of Anticoagulant Therapy. Innov. Surg. Sci..

[25] Mihai S., Codrici E., Popescu I.D., Enciu A.M., Albulescu L., Necula L.G., Mambet C., Anton G., Tanase C. (2018). Inflammation-Related Mechanisms in Chronic Kidney Disease Prediction, Progression, and Outcome. J. Immunol. Res..

[26] Ellulu M.S., Patimah I., Khaza’ai H., Rahmat A., Abed Y. (2017). Obesity and Inflammation: The Linking Mechanism and the Complications. Arch. Med. Sci..

[27] Cheng C., Yan Y., Zhang Q., Guo W. (2021). Effect of Chronic Kidney Disease on Total Knee Arthroplasty Outcomes: A Meta-Analysis of Matched Control Studies. Arthroplasty.

[28] Aggarwal V.A., Sambandam S.N., Wukich D.K. (2022). The Impact of Obesity on Total Knee Arthroplasty Outcomes: A Retrospective Matched Cohort Study. J. Clin. Orthop. Trauma.

[29] Valkanoff T.A., Kline-Simon A.H., Sterling S., Campbell C., Von Korff M. (2012). Functional Disability Among Chronic Pain Patients Receiving Long-Term Opioid Treatment. J. Soc. Work Disabil. Rehabil..

[30] Darnall B.D., Stacey B.R., Chou R. (2012). Medical and Psychological Risks and Consequences of Long-Term Opioid Therapy in Women. Pain Med..

[31] Vadeghani A.T., Grant M., Forget P. (2024). Perioperative Pain Management Interventions in Opioid User Patients: An Overview of Reviews. BMC Anesthesiol..

