# Supplementary material for: Postoperative Complications Following Total Knee Arthroplasty in Patients with Chronic Lymphocytic Leukemia
Source: J Clin Med. 2026 Aug 5;15(15):6078. doi: 10.3390/jcm15156078 (PMC13467093; doi:10.3390/jcm15156078)
Supplement: Supplementary file 1 [file jcm-15-06078-s001.zip › jcm-4379375-supplementary.pdf]

Table S1. Codes Used to Define Cohorts, Covariates, Procedures, and Postoperative Outcomes in TriNetX

Group	Code(s)
Abnormalities of Gait and Mobility	ICD-10-CM code R26
Acute Blood Loss Anemia	ICD-10-CM code D62
Acute Kidney Injury	ICD-10-CM code N17
Anticoagulant Use	VA code BL110
Arthrofibrosis	ICD-10-CM code M24.661, ICD-10-CM code M24.662, ICD-10-CM code M24.669
Atelectasis	ICD-10-CM code J98.11
BTK Inhibitors	ATC code L01EL
Chemotherapy	TNX Curated code 1002
Chronic Kidney Disease	ICD-10-CM code N18
Chronic Lymphocytic Leukemia	ICD-10-CM code C91.1
Chronic Obstructive Pulmonary Disease	ICD-10-CM code J44
Diabetes	ICD-10-CM code E08-E13
Emergency Department Visits	Visit: Emergency
Essential (Primary) Hypertension	ICD-10-CM code I10
Heart Failure	ICD-10-CM code I50
Long-Term Use of Anticoagulants and Antithrombotics/Antiplatelets	ICD-10-CM code Z79.0
Long-Term Use of Opiate Analgesic	ICD-10-CM code Z79.891
Lower Extremity Deep Vein Thrombosis	ICD-10-CM code I82.4

Manipulation Under Anesthesia	CPT code 27570
Mechanical Complications	ICD-10-CM code T84.0
Overweight and Obesity	ICD-10-CM code E66
Periprosthetic Fracture	ICD-10-CM code M97.1
Periprosthetic Joint Infection	ICD-10-CM code T84.53, ICD-10-CM code T84.54
Pneumonia	ICD-10-CM code J12, ICD-10-CM code J13, ICD-10-CM code J14, ICD-10-CM code J15, ICD-10-CM code J16, ICD-10-CM code J17, ICD-10-CM code J18
Postoperative Infection	ICD-10-CM code T81.4
Postoperative Pain	ICD-10-CM code G89.18
Prosthetic Dislocation	ICD-10-CM code T84.02
Readmission	Visit: Inpatient Encounter
Reduced Mobility	ICD-10-CM code Z74.0
Revision Total Knee Arthroplasty	CPT code 1005095, ICD-10-PCS code 0SWC, ICD-10-PCS code 0SWD
Stem Cell Transplantation	TNX Curated code 1005
Tobacco Use	ICD-10-CM code Z72.0
Total Knee Arthroplasty	CPT code 1005073, ICD-10-PCS code OSRC, ICD-10-PCS code OSRD
Transfusion	CPT code 1012085
Venetoclax	RxNorm code 1747556

BTK = Bruton tyrosine kinase
